# Enhanced Efficacy of High Dose Oral Vancomycin Therapy in *Clostridium difficile* Diarrhea for Hospitalized Adults Not Responsive to Conventional Oral Vancomycin Therapy: Antibiotic Stewardship Implications

**DOI:** 10.3390/jcm7040075

**Published:** 2018-04-10

**Authors:** Burke A. Cunha, Julia Sessa, Sharon Blum

**Affiliations:** 1 Infectious Disease Division, NYU Winthrop Hospital, Mineola, NY 11501, USA; 2 State University of New York, School of Medicine, Stony Brook, NY 11794, USA; 3 Department of Pharmacy, Winthrop-University Hospital, Mineola, NY 11501, USA; jsessa@winthrop.org (J.S.); Sblum@nyuwinthrop.org (S.B.)

**Keywords:** oral vancomycin, *C. difficile* diarrhea, oral vancomycin dosing regimens, metronidazole ineffectiveness

## Abstract

Current therapy of *Clostridium difficile* diarrhea (CDD) is problematic. Optimal treatment for CDD remains oral vancomycin, but there is little data on oral vancomycin dosing regimens. The objective of this *C. difficile* diarrhea study was to compare the efficacy of “high dose” vancomycin, 500 mg (PO) q6h, as sole treatment and in those who after 72 h failed to respond to conventional doses of oral vancomycin, 125–250 mg (PO) q6h. Hospitalized adults with CDD were evaluated by various oral vancomycin regimens, i.e., a conventional dose group (125–250 mg (PO) q6h), a “high dose escalation” dose group (250 mg → 500 mg (PO) q6h), and a “high dose” group (500 mg (PO) q6h). Oral vancomycin treatment groups were compared by time to improvement, i.e., decrease in >50% of watery stools/day and duration of therapy. The high dose escalation and high dose oral vancomycin groups showed the most rapid resolution of diarrhea. There was marked decrease in stools/day after “high dose” vancomycin escalation from conventional dosing, i.e., 250 mg (PO) q6h → 500 mg (PO) q6h. This study demonstrated that “high dose” escalation or initial high dose oral vancomycin, i.e., 500 mg (PO) q6h was the most efficacious regimen for CDD.

## 1. Introduction

*Clostridium difficile* infection (CDI) is an imprecise term that does not describe the severity of infection. Multiple risk factors are now appreciated; among these, advanced age has been found to be important. Patients ≥ 65 years of age represented 92% of *C. difficile* related hospital stays in the United States. In the United States, 93% of deaths from *C. difficile* colitis occurred in persons ≥65 years of age [1,2,3,4,5,6,7,8,9,10,11]. Some antimicrobials alter gut flora and induce toxin production, resulting in *C. difficile* diarrhea and/or colitis [1,2,3]. Some antibiotics are major risk factors for *C. difficile* diarrhea, e.g., clindamycin or β-lactams. Importantly, most antibiotics are rarely associated with *C. difficile*, e.g., aminoglycosides, aztreonam, colistin, polymyxin B, quinupristin/dalfopristin, tetracyclines, tigecycline, macrolides, sulfamethoxazole-trimethoprim, daptomycin, linezolid, telavancin, dalbavancin, oritavancin, fosfomycin, nitrofurantoin, and chloramphenicol. In our experience, over the last four decades, clindamycin, cephalosporins, and, less commonly, fluoroquinolones (especially ciprofloxacin) have an increased risk of *C. difficile* compared to other antimicrobial agents. The risk of *C. difficile* is greatly increased with concomitant administration of proton pump inhibitors (PPIs). Exposure to clindamycin or β-lactams not only increases *C. difficile* risk during therapy but also for three months following therapy [12]. Data from the Centers for Disease Control and Prevention Emerging Infections Program in 2010 reported that hospitalization was a major risk factor for development of *C. difficile* as 94% of cases were associated with healthcare including recently discharged patients, outpatients, and those in long-term care facilities [13]. Other *C. difficile* risk factors include some antipsychotic agents, stool softeners/laxatives, and some chemotherapeutic agents [14,15]. Some populations i.e., those with kidney disease, human immunodeficiency virus (HIV), and solid organ transplants all have relatively higher incidences of *C. difficile* [16,17,18,19]. In contrast, enteral feeds are protective against *C. difficile* as are some antibiotics, e.g., doxycycline or tigecycline [20,21,22].

Current clinical practice guidelines for *C. difficile* published by the Society for Healthcare Epidemiology of America (SHEA) and the Infectious Diseases Society of America (IDSA) recommend initial treatment of mild to moderate disease with (diarrhea) metronidazole 500 mg (PO) q8h or vancomycin 125 mg (PO) q6h for 10–14 days. For the treatment of severe disease (colitis), a combination of metronidazole (IV/PO) and vancomycin 500 mg (PO) q6h, is recommended [23]. Metronidazole, the mainstay of treatment of *C. difficile* (colitis), is considered suboptimal therapy for *C. difficile* diarrhea (vs. oral vancomycin). There is no general agreement on optimal treatment for patients who do not respond to guideline recommendations and only limited published data on the treatment of such patients [24,25,26,27,28].

Vancomycin was the first effective antimicrobial for the treatment of *C. difficile* and the antibiotic standard against which all successive therapies have been compared. Vancomycin is a unique glycopeptide: when administered orally, vancomycin essentially is not absorbed, i.e., ingestion does not result in appreciable serum levels. Vancomycin concentrates intra-luminally in the colon, making it ideal for the treatment of *C. difficile* diarrhea (CDD), a mucosal process [24]. This contrasts with colitis, a transmucosa where metronidazole is effective. In the era of antimicrobial stewardship programs (ASP), CDD as well as treatment failures remain important concerns. Relapse rates with conventional oral vancomycin dosing, 125 mg (PO) q6h, have been reported to be as high as 30%, prompting a search for optimal oral vancomycin dosing regimens for CDD [25]. In our own experience over three decades, conventionally dosed vancomycin, 125 mg (PO) q6h, has often failed to achieve rapid clinical improvement of *C. difficile* diarrhea. Accordingly, this has resulted in our approach of using 250 mg (PO) q6h as the initial treatment regimen for *C. difficile* presenting as diarrheal disease (CDD). It also has been our practice to increase vancomycin dosing to 500 mg (PO) q6h if there is lack of clinical improvement (≥50% decrease in the number/volume of watery stools/day) after 72 h following treatment with our standard vancomycin regimen, i.e., 250 mg (PO) q6h. 

Our primary objective was to evaluate the enhanced therapeutic efficacy of oral vancomycin high dose escalation to 500 mg (PO) q6h for those with CDD who failed to improve substantially (≥50% decrease in the number/volume of watery stools/day) after three days of vancomycin 250 mg (PO) q6h therapy. The secondary objective of this retrospective study included assessing the efficacy of “high dose” vancomycin 500 mg (PO) q6h as the primary treatment dose and to examine concomitant metronidazole utilization.

Rapid resolution of CDD is best for the patient, decreases potential exposure to other patients, allows earlier discontinuance of CDD precautions, and permits earlier discharge. 

## 2. Methods

NYU Winthrop Hospital is a 600-bed university teaching hospital located in Long Island, New York. This was a quality assurance retrospective study utilizing electronic medical records (EMR) to review hospitalized adults with CDD from January 2015 to December 2016. Patients were identified utilizing MedMined^®^, an online system to identify patients with otherwise unexplained acute watery diarrhea who tested positive for *C. difficile* toxin via stool polymerase chain reaction (PCR). All data on patients in the study were appropriately de-identified. There is no institutional protocol to treat *C. difficile* and treatment is at the discretion of the physician. 

Hospitalized adults with acute onset watery diarrhea who were stool PCR positive for *C. difficile* treated with oral vancomycin were eligible for inclusion. Excluded were patients with fecal collection bags, immunocompromised patients, or those with incomplete documentation regarding diarrhea as we were unable to determine number of stools and days until soft stool to evaluate treatment response.

The objective of this retrospective study was to assess the efficacy of “high dose” vancomycin, 500 mg (PO) q6h, in patients with CDD failing to substantially improve (decrease ≥50% of liquid stools/day) after 72 h. Clinical improvement was defined as a ≥50% decrease in the number/volume of watery stools/day. Clinical resolution was defined as days to soft/formed stools.

## 3. Results

**Figure 1:**
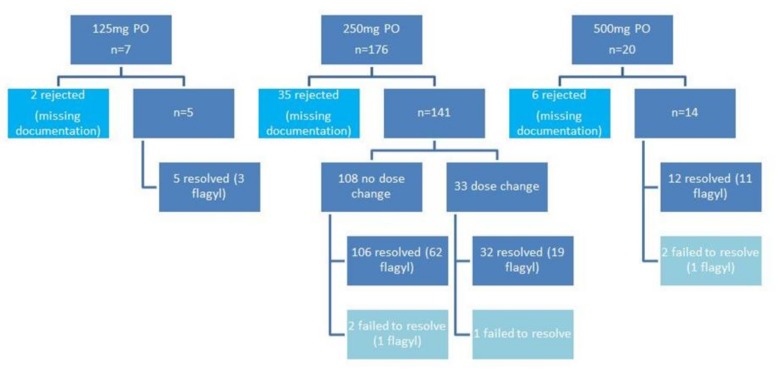
Patient flowchart.

Over a 24 month period, 203 non-ICU patients with acute watery stools positive for *C. difficile* toxin by PCR were identified. Of these, 160 were eligible for analysis and were divided into three groups, i.e., conventional dosing group, high dose escalation group, and high dose group as primary treatment. See Figure 1 for further description of patient flow.

### 3.1. Conventional Dosing Group

Out of the 160 patients included in the study for analysis, 108 patients were treated with vancomycin 250 mg (PO) q6h for the entire course of therapy and five patients were treated with 125 mg (PO) q6h for the entire course of therapy. The mean age in this group was 69.6 years (range: 20–102) and 57% were female. In this group, the mean number of watery stools upon diagnosis was 5.6/day (range: 2–17/day); after 72 h of therapy, it decreased to 2.68/day (range: 0–9/day). Clinical resolution occurred at 5.08 days (range: 2–15 days). A total of 65 patients in this group (58%) were concurrently treated with metronidazole. In this patient group, the average number of vancomycin doses was 53.4 (range: 12–205) and the average duration of therapy was 14.2 days (range: 4–54 days). Included were two patients who did not achieve clinical resolution including one on concurrent metronidazole.

### 3.2. High Dose Escalation Group

Of the 160 patients included in the study, 33 patients underwent “high dose” escalation after failing to respond to initial conventional dose vancomycin regimens within three days. In this patient population, the mean age was 68.4 (range 20–97) and 48% were female. At presentation, the mean number of watery stools was 6.45/day (range: 4–15/day). After 72 h of initial therapy with 250 mg (PO) q6h, the mean number of watery stools was 5.5/day (range: 3–12 days). As a result of lack of clinical improvement, the dose was increased to 500 mg (PO) q6h and the number of watery stools 72 h after dose escalation was 2.5/day (range: 0–9/day). Clinical resolution occurred at day 10 (range: 4–27) after initiation of vancomycin therapy and 4.2 days (range: 1–6 days) after the first dose of 500 mg was administered. Included were 19 patients also treated with metronidazole at the initiation of therapy (58%) and two patients discontinued metronidazole post-dose escalation. The average number of vancomycin doses was 64.4 (range: 21–180), of which 21.9 (range: 4–78) were 250 mg and 37.3 (range: 13–98) were 500 mg. The average total duration of therapy was 17.97 days (range: 9–53 days). The average length of therapy after transition to “high dose” oral vancomycin was 11.5 days (range: 5–47 days). Only one patient did not achieve clinical resolution after switching to 500 mg PO q6h (Table 1).

### 3.3. High Dose Group

**Figure 2:**
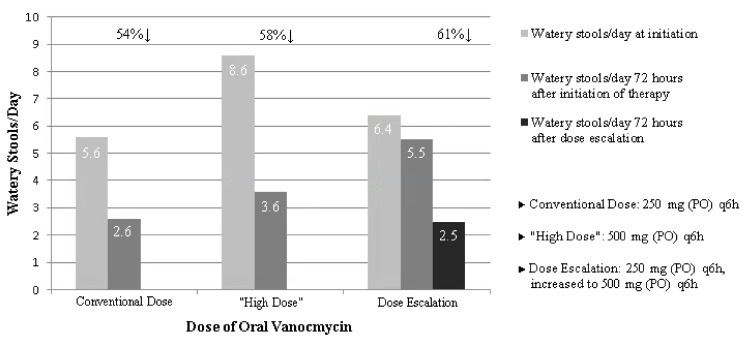
Number of *C. difficile* watery stools/day at onset and response to oral vancomycin therapy regimens.

**Figure 3:**
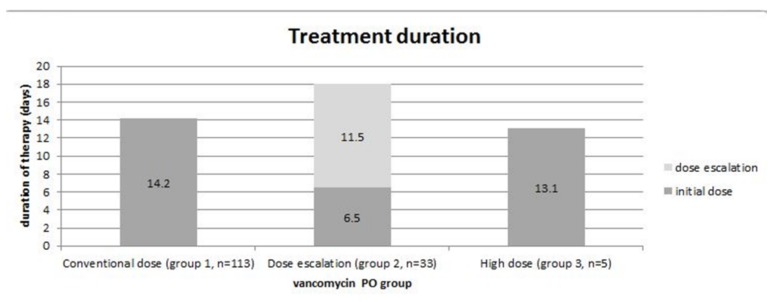
Duration of oral vancomycin therapy by vancomycin dose.

Out of the 160 patients included in this study, 14 patients were treated with vancomycin 500 mg (PO) q6h for the entire course of therapy. The mean age was 64.4 (range 54–94) and 50% were female. The mean number of watery stools/day at presentation was 8.8/day (range: 5–17/day) and after 72 h of therapy it was 3.6/day (range: 1–8/day). Clinical resolution was noted at 5.4 days (range: 3–15 days). Included were 11 patients concurrently treated with metronidazole (79%). In this group, the average number of vancomycin doses was 53 (range: 8–114) and the average duration of therapy was 13.1 days (range: 6–31 days). Only two patients did not achieve clinical resolution. A summary of results is provided (Figure 2 and Figure 3).

## 4. Discussion

The results of this limited retrospective quality assurance study showed that, in patients failing to achieve rapid clinical improvement of CDD after 72 h with conventional vancomycin dosing, i.e., 125–250 mg (PO) q6h, “high dose” vancomycin 500 mg (PO) q6h was a highly effective treatment option. Consistently, “high dose” vancomycin therapy resulted in a rapid decrease in number of watery stools/day. Treatment for CDD failing to respond to standard vancomycin therapy has consistently been a dilemma for clinicians at our institution. For years, we have observed a high degree of efficacy with “high dose” vancomycin 500 mg (PO) q6h, and we were able to demonstrate this via formal review and analysis. We demonstrated a 97% response rate to vancomycin 500 mg PO q6h in patients who failed to response to 250 mg q6h.

There are a limited number of studies, all of which involve a small number of patients, specifically analyzing the efficacy of “high dose” vancomycin in the treatment of CDD. All show rapid rates of clinical resolution, ranging from 92% to 100%, with an average time to resolution of three days [26,27,28]. 

The sooner a clinical resolution of diarrhea is achieved, the earlier infection control precautions for *C. difficile* may be discontinued. Compared to hospital costs of lengths of stay (LOS) attributable to *C. difficile*, the cost of “high dose” oral vancomycin therapy is negligible. 

An interesting finding of this study was that regarding duration of treatment. Provided the patient’s diarrhea has resolved, national guidelines recommend a 10- to 14-day course of therapy, i.e., 125–250 mg (PO) q6h; however, we found practitioners often continued therapy beyond the recommended duration despite no data to support the continued oral vancomycin beyond clinical resolution. 

From the perspective of an antimicrobial stewardship program (ASP), it was interesting to note the role of concomitant metronidazole. Metronidazole was frequently used in all patient groups. However, concomitant metronidazole did not offer any additional benefit independent of vancomycin dose. 

Although this study was intended to limit confounding variables, there were limitations to this study. The main limitations were relatively small sample size and a retrospective study design. Accurate stool documentation relied entirely on the accurate documentation of the patient’s nurse; treatment choice dose and duration was entirely dependent on the patient’s physician. From an ASP perspective, an institutional protocol would provide improved uniformity of outcomes demonstrated by our data as well as the elimination of the use of concomitant metronidazole in these patients with CDD, not *C. difficile*. 

This retrospective quality assurance study was conducted to assess the efficacy of “high dose” oral vancomycin as an effective treatment option in patients with CDD (not colitis) who failed to substantially improve with conventionally dosed oral vancomycin regimens after 72 h. Out of 203 patients, 33 underwent “high dose” escalation and 32 demonstrated resolution. The more rapidly a medication is able to provide clinical improvement/resolution, the sooner the patient may be discharged from the hospital, i.e., decreased LOS. Because oral vancomycin is poorly absorbed in the gastrointestinal tract, “high dose” oral vancomycin escalation is safe and highly effective. Large-scale prospective, randomized, control trials are necessary to assess the optimal dose for treating patients who failed to improve on oral vancomycin therapy. Nevertheless, our single center experience provides support for “high dose” vancomycin escalation or as initial therapy [25]. Importantly, metronidazole did not enhance the efficacy of oral vancomycin therapy independent of oral vancomycin dose [25,29,30]. Our data supports our clinical experience that preferable initial therapy of CDD should be treated with vancomycin (PO). The role of metronidazole should be relegated to *C. difficile* colitis. 

From an infection prevention and ASP perspective, rapid resolution of CDD has several benefits for the patient and hospital. Faster resolution is better for the patient, decreases potential exposure of other patients to CDD, allows earlier discontinuance of precautions, and earlier discharge.

## Figures and Tables

**Table 1:** Oral vancomycin regimens for *C. difficile* diarrhea.

	Conventional Dosing (*n* = 113)	High Dose (*n* = 14)	Dose Escalation (*n* = 33)
Age (mean, years)	69.6	64.4	68.4
Female (%)	57	50	48
Concurrent metronidazole (*n*, %)	65, 58	11, 79	19, 58
Number of stool/day at diagnosis (mean)	5.60	8.80	6.45
Number of stool/day at 72 h (mean)	2.68	3.60	5.50
Number of stool/day at 72 h following dose escalation (mean)	N/A	N/A	2.5
Days to clinical resolution (mean)	5.08	5.40	10.00
Days to clinical resolution from dose escalation (mean)	N/A	N/A	4.2
Total vancomycin doses (mean)	53.4	53.0	64.4 (250 mg: 21.9; 500 mg: 37.3)
Total days of vancomycin therapy (mean)	14.20	13.10	17.97
Total days of vancomycin therapy from dose escalation (mean)	N/A	N/A	11.5
